# Interpersonal Synchronization, Motor Coordination, and Control Are Impaired During a Dynamic Imitation Task in Children With Autism Spectrum Disorder

**DOI:** 10.3389/fpsyg.2018.01467

**Published:** 2018-09-03

**Authors:** Jean Xavier, Soizic Gauthier, David Cohen, Mohamed Zahoui, Mohamed Chetouani, François Villa, Alain Berthoz, Salvatore Anzalone

**Affiliations:** ^1^Département de Psychiatrie de l'Enfant et de l'Adolescent, AP-HP, Hôpital Pitié-Salpêtrière, Paris, France; ^2^Sorbonne Université, Institut des Systèmes Intelligents et de Robotique, CNRS UMR 7222, Paris, France; ^3^CRPMS, EA 3522, Université Paris Diderot, Sorbonne Paris Cité, Paris, France; ^4^Equipe Berthoz, Collège de France, Paris, France; ^5^Laboratoire CHArt-THIM, EA4004, Université Paris 8, Saint-Denis, France

**Keywords:** imitation, interpersonal synchronization, motor coordination, motor control, autism spectrum disorder, developmental coordination disorder

## Abstract

**Background:** Impairments in imitation abilities have been commonly described in children with autism spectrum disorder (ASD). How motricity in interpersonal coordination impacts imitation, during long lasting semi-ecological conditions, has not been carefully investigated.

**Methods:** Eighty-five children and adolescents (39 controls with typical development, TD; 29 patients with ASD; 17 patients with developmental coordination disorder, DCD), aged 6 to 20 years, participated to a behavioral paradigm in which participants, standing and moving, interacted with a virtual tightrope walker standing and moving as well. During the protocol, we measured automatically and continuously bodily postures and movements from RGB sensor recording to assess participants' behavioral imitation.

**Results:** We show that (1) interpersonal synchronization (as evidenced by the synchrony between the participant's and the tightrope walker's bars) and (2) motor coordination (as evidenced by the synchrony between the participant's bar and its own head axis) increased with age and were more impaired in patients with ASD. Also, motor control as evidenced by the movement angle standard deviations of participants' bar and head were significantly impaired in ASD compared to TD or DCD.

**Conclusion:** Interpersonal synchronization and motor coordination during ecological interaction show both subtle impairment in children with ASD as compared to children with TD or DCD. These results questioned how motricity mature in terms of motor control and proprioception in children with ASD.

## Introduction

Imitation plays a critical role in the development of intersubjectivity. It represents a key milestone in early communication (Gopnik and Meltzoff, 1993; Nadel and Potier, 2002; Rogers et al., 2005), a bedrock on which social cognition is built (Meltzoff, 2007) and a prerequisite of the self (Meltzoff, 2005). From an evolutional and developmental perspective, increased self-other distinction occurs through imitation, from motor mimicry (copying the style or the form of bodily movements of a model), to coordination and emulation (sharing the goals or the results of an action, but not the means used to achieve the goals), and to true imitation (reproduction of both the goals and the means of the observed actions) (de Waal, 2008). Motor imitation is a shared experience underlined by the perception that others are “like me” through an innate coupling between observation and execution of human actions, i.e., the existence of a structural congruence between the perception of others and one's own behavior (Meltzoff and Decety, 2003). Spontaneous motor imitation between children reveals a playful dynamic, driven by repetition, challenging the visual-spatial abilities of children (Xavier et al., 2013). It also requires continuous partner reciprocity involving synchrony rooted in rhythmic interpersonal coordination, which is promoted, during joint actions, by dynamic similarities in terms of motor signature (Xavier et al., 2017).

Impairments in imitation abilities have been described in neurodevelopmental disorders such as developmental coordination disorder (DCD) and autism spectrum disorder (ASD). Children with DCD display motor incoordination and visual spatial processing deficits (Mazeau, 2000) which may affect their imitation abilities (Green et al., 2006; Werner et al., 2012). Autism known, as a spectrum disorder because it refers to a wide range of conditions, is defined by impaired social communication function and the presence of restricted, repetitive patterns of behavior or interest as core symptoms (American Psychiatric Association, 2013). Children with ASD can also display visual spatial difficulties as well as impairments in motor coordination, control of posture, performance of gestures and complex movement sequences (Henderson et al., 1992; Ghaziuddin and Butler, 1998; Jansiewicz et al., 2006; Fournier et al., 2010; MacNeil and Mostofsky, 2012). They may also manifest lower interpersonal synchrony (Marsh et al., 2013; Fitzpatrick et al., 2016) and developing deficits in control of movements (Mari et al., 2003; Rinehart et al., 2006; Dowd et al., 2012; Gowen and Hamilton, 2013). Thereon, Trevarthen and Delafield-Butt (2013) support the presence of a disturbance of primary prospective motor control of expressive action in ASD, affecting future social expectation and understanding. Many studies have linked ASD to problems with imitation (for recent reviews see Rogers and Williams, 2006; Nadel, 2014; Vivanti and Hamilton, 2014; Williams, 2018) which could be underlined by a dysfunction in the mirror neuron system (Williams et al., 2001). According to Goldman (2006), this system is involved in simulation theory, as low level simulation-based mindreading, permitting the observer to perform an action like the one being watched, thereby getting the observer “into the mental shoes” of the observed. In this way, problems in the functioning of mirror neuron system could be involved in social cognitive impairments in ASD (Wilkinson and Ball, 2012).

However, considering the heterogeneity of ASD (Xavier et al., 2015) as well as the heterogeneity of imitation performances in ASD (Rogers et al., 2010; Vivanti et al., 2011; Salowitz et al., 2013), inconsistencies and conflicting results exist regarding the nature of this deficit and the presence of a general imitation deficit specific *per se* in ASD (Vanvuchelen et al., 2011; Vivanti and Hamilton, 2014). Furthermore, the diversity of ages, tasks, and developmental levels of children participating in different studies makes comparison across findings difficult. To date, research has failed to clarify whether differences in imitation reflect a deviance (Rogers et al., 2003) or a delay (Young et al., 2011) from typical development.

Overall, studies found that, in comparison to typically developing (TD) children, children with ASD often have a low propensity to imitate (Vivanti et al., 2014), imitate less precisely, and appear to have more difficulties early in development than later on (Williams et al., 2004). Furthermore, in comparison to TD children, this population was found to display similar performances when imitated actions have a visual goal or meaning (Rogers et al., 1996; Gowen, 2012), and lower performances when imitation tasks are goal-less or meaningless (Williams et al., 2004; Hobson and Hobson, 2008; Rogers et al., 2010; Cossu et al., 2012; Nielsen et al., 2013) and when imitation tasks involve body postures or kinematics (Gowen, 2012; Vivanti and Hamilton, 2014). Contrary to performances in emulation tasks for which autistic participants tend to be proficient, it is likely that children with ASD engage less in mimicry behavior (the means of the action) than TD peers (Edwards, 2014). This suggests a failure to use the kinematic details of the action such as its amplitude, speed, or trajectory (Bekkering et al., 2000; Rumiati and Tessari, 2002; Carpenter et al., 2005; Wild et al., 2010). Several factors related to imitation deficits include poor visual encoding, self-other mapping problems (Williams et al., 2001), and motor, praxis or sensorimotor related disturbances (for a review see Vivanti et al., 2013).

Tasks involved in the abovementioned studies did not investigate imitation involving body movements while an individual is spontaneously interacting with another. Furthermore, comparison to other groups such as children with DCD is infrequent. The developmental aspects of motor imitation in children with ASD and DCD have not yet been addressed in a semi-ecological task such as Thirioux et al. 's experimental setup (2009). This spontaneous motor imitation task during an interaction between a participant and a tightrope walker avatar was first designed to investigate own-body-transformation (OBT) abilities of the participants. In a previous study comparing children with ASD or DCD and typically developing children (TD), we found that (1) OBT in a spatial environment was not possible in this experience before age 11; (2) yet it was possible later for patients with ASD although delayed compared with TD children (Gauthier et al., 2018).

Here we explore behavioral imitation abilities in terms of interpersonal synchronization, motor coordination and control by means of this interaction paradigm. To get a better understanding of imitation difficulties in children with ASD, we explored the potential alteration of the development of behavioral imitation abilities in children with ASD in comparison with DCD children and TD control children. ASD and DCD have in common motor and visual spatial difficulties. Therefore, comparing these two pathological groups offered the opportunity to disentangle the contribution of visual-spatial and motor coordination impairments in motor imitation difficulties.

The following hypotheses were made: (1) within the TD group, there will be a positive developmental/age effect on interpersonal synchronization, motor coordination and control. This developmental effect will be explored in the ASD and DCD groups. (2) When compared to TD children, children with ASD will show significant impairments in terms of interpersonal synchronization, motor coordination and control. (3) When compared to TD children, children with DCD will also show significant impairments in terms of interpersonal synchronization, motor coordination and control, but to a lesser extent compared to ASD Children.

## Method

### Participants

A total of 85 children and adolescents, aged 6 to 19 years, were recruited in the department of Child and Adolescent Psychiatry of the Pitié-Salpêtrière University hospital. Given the lack of previous developmental study, we included a large control group with typical development. Inclusion criteria for the patients were (1) a diagnosis of DCD or a diagnosis of ASD; (2) the cognitive ability to understand the imitative task which was checked during a motor imitation game with the clinicians involved in the study. Exclusion criteria were ongoing medical conditions (e.g., seizures, sensory deficit) and severe language impairment that can be comorbid with ASD and DCD. For each patient, the diagnoses were based on all available information (including direct interviews, family history data, treatment records) and computed according to the Diagnostic and Statistical Manual of Mental Disorders-Fifth Edition criteria (American Psychiatric Association, 2013). Each patient was also given a series of clinical assessments: the Autism Diagnostic Interview-Revised (ADI-R) was used to score autism core symptoms (Lord et al., 1994); the cognitive quotient was ascertained by using the WISC-IV (Wechsler Intelligent Scale for Children-IV), or the Psycho-educational Profile—Third Edition (PEP-3) (Schopler et al., 1990) according to age. Developmental age was calculated on this basis. Each child with ASD was individually matched, according to developmental age, with a healthy TD child using chronological age, assuming that for TD children, chronological and developmental age where equal. Children with DCD were evaluated during a psychomotor assessment that included quantitative testing (e.g., the Movement Assessment Battery for Children, M-ABC) (Henderson et al., 1992) performed by an occupational therapist. The TD children were recruited via the staff of the child and adolescent psychiatry department of the Pitié-Salpêtrière hospital. They were matched for age with the patients. Including written informed parental consent, the study was specifically reviewed and approved by an ethics committee, the CERES (*Comité d'Ethique de la Recherche en Santé*) [N° IRB: 20150700001072]. Among the 85 recruited participants, 5 (4 with ASD and 1 with DCD) did not complete the experiment and were not included in the analysis: 3 participants could not lean like the tightrope walker while not walking like the tightrope walker; 1 participant could partially perform the tasks but decided to stop it too early; 1 participant could not perform the tasks because he wanted to fight with him and to attack his eyes. Demographics and clinical characteristics of the 80 participants with exploitable data are given in Table 1.

**Table 1:** Main characteristics of the participants.

	**ASD (*N* = 26)**	**DCD (*N* = 15)**	**TD (*N* = 39)**
Chronological age, mean (±SD)	12.65 (3.66)	12.17 (3.38)	11.95 (4.08)
Male/Female	21/5	9/6	23/16
**WISC-4, mean (**±**SD)**			
Verbal comprehensive index	94.33 (30.60)	98.69 (17.54)	Non-relevant
Perceptual organization index	88.30 (28.69)	92.92 (17.70)	
Working memory index	80.57 (25.73)	85.72 (17.89)	
Processing speed index	73 (17.23)	84.54 (17.81)	
Developmental age (IQ^*^ age /100)	11.75 (5.08)	11.61 (3.23)	
**ADI-R scores, mean (**±**SD)**			
Social impairment	14.87 (6.86)	Non-relevant	Non-relevant
Verbal communication	10.04 (5.35)		
Restricted, repetitive behaviors	3.83 (3)		

*ASD, Autism spectrum Disorder; DCD, developmental coordination disorder; TD, children with typical development; ADI-R, Autism Diagnostic Interview—Revised version; WISC-4, Wechsler Intelligence Scale for Children (version 4)*.

### The tightrope walker paradigm

**Figure 1:**
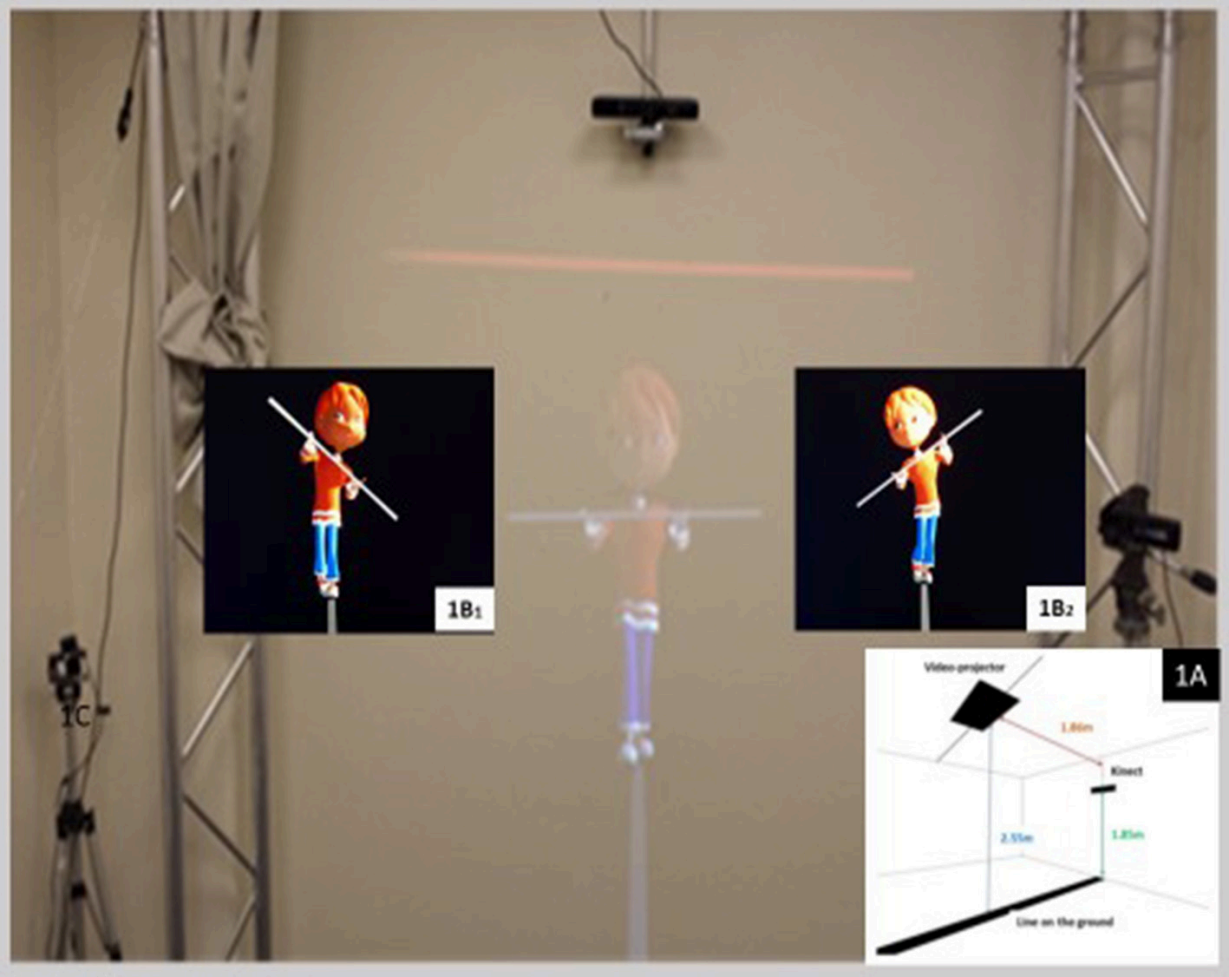
Principles and set-up of the experiment: **(1A)** Schematic illustration of the experimental room with the projection on the wall of the tightrope walker avatar; **(1B**_**1,2**_**)** Tightrope walker avatar's head and bar inclinations in the front-facing orientation.

The tightrope walker paradigm (Thirioux et al., 2009) is an experimental setup designed to test the ability to change spatial viewpoints i.e., own-body transformations, during a spontaneous motor imitation task (Figure 1). We adapted the paradigm of the “*funambule*” tightrope walker to children by adjusting the size and giving it a cartoon aspect of a child. We developed a 3D animation where a 3D character is walking on a rope and hold a bar in front of him (Figure 1). The 3D animation was developed under Unity—a 3D engine used for virtual reality. The application runs on a PC under Windows 7. The natural movements of the 3D character come from a series of motion capture with the 12 cameras of VICON system. The animated tightrope walker (TW) walking on a rope was displayed life-sized by a rear-projector onto a large screen (2 ^*^ 2 m). It was 0.81 meter-high when standing in the middle part of his rope, 1.13 meter-high when he was the “closest” to the participant. To mimic everyday social encounters and to reinforce interactions giving participants the impression to act in the same spatial environment as the TW, participants stood on a black line (2 /10 cm; length/width) which prolonged on the ground the avatar's rope on the screen (Figure 1A). Before the movie started, we asked participants to find a comfortable position, legs slightly apart and not to shift from their position in response to the moves of the TW. Participants held a wooden bar (length: 1 m) horizontally in front of them. In order to reinforce both the interaction with the TW and the ecological features of the task, the TW's forward and backward movement's duration were randomized.

### Protocol and tasks

First, the tightrope walker (TW) was shown in a front-facing orientation, standing with his right foot in front of the left on the rope for the first 30 s. Then, during 7 following trials, numbered from 1 to 7, the TW, walking successively either forward or backward, was alternatively shown in two orientations: (i) a front-facing orientation when the TW walks forward and a back-facing orientation when, the TW walking backward, the participants saw it from his back. While walking, for each orientation, the TW executed lateral tilts with his bar in random order either to his right or his left (Figure 1,B_1,2_), with a maximum amplitude of 51° (mean amplitude: 44°) and a maximum duration of 3.2 s (mean duration: 2.7 s). Each trial lasted 35.7 s and was composed of 7 TW's tilts. For half of the participants in each group, the first trial presents the TW front-facing and for the other half the first trial presents the TW back-facing. Participants were instructed to observe the tilts of the TW and to lean when he was leaning. Participants were also asked not to walk even when the TW was walking and to stand still when he was.

### Data recording and metrics

**Figure 2:**
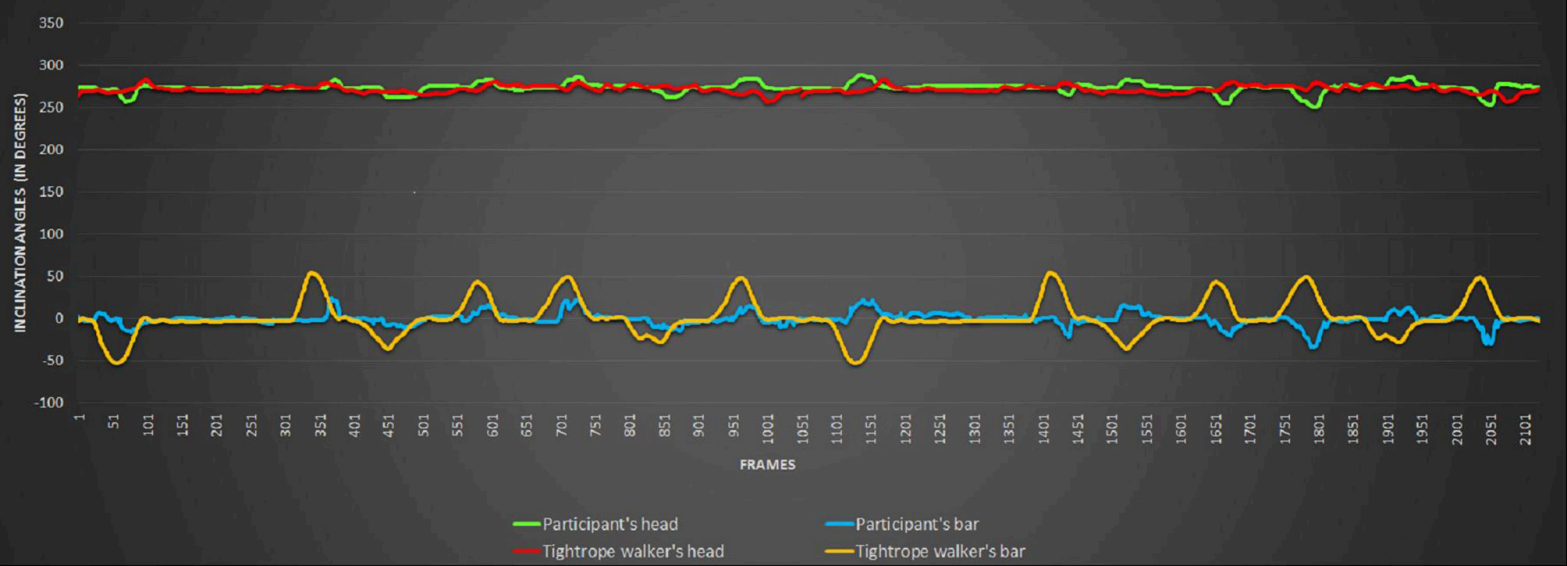
The inclination angles of the head and of the bar in the frontal plane were recorded for the participant and the avatar. The example shows the recording of the inclination in degrees of the participant's head and bar and tightrope walker avatar head and bar, according to the chronological progress of the frames.

Participants' bodily postures and movements in the frontal plane were automatically and continuously recorded for offline analysis and labeling. We used a RGB sensor (KINECT), located in front of them, on the wall above the TW, at a height of 1.85 m. The KINECT captured the figure of the participant at a mean rate of about 25 frames per second. The information contained by each frame was accessible through a comma-separated values (csv) file. For each frame, the participant's posture and the TW's posture were recorded as well as the timing of the frame, the participant's and the TW's bar and head inclinations were measured in degrees (Figure 2).

The interpersonal synchronization defined as the synchrony between the participant's and the tightrope walker's bars was assessed using the correlation between the bar angles of the TW and that of the participants. For each participant, the motor coordination and the motor control were respectively measured evaluating (i) the correlation between the participant's bar and its head axis angle; (ii) the movement angle's standard deviations of participant's bar and head.

### Statistical analysis

Statistical analyses were performed using R Software, Version 2.12.2. For every test, the level of significance, alpha, was fixed at 5%. To assess the variables of interests, we assessed the following dependent variables using Generalized Linear Mixed Model (GLMM; lme4 package): correlation coefficient between the participant's bar and the tightrope walker's bar angles, correlation coefficient between the participant's bar and its head axis angles, standard deviation of the participant's bar movements, standard deviation of the participant's head. The following explicative variables were entered in each model: developmental age, type of group (TD vs. DCD vs. ASD), and the trial number (1 to 7). For each dependent variable, the normal distribution was checked. Variable transformations were conducted to reach normalization when needed.

## Results

We have divided the results for two age groups under and above 12 years, based on the results of Gauthier et al. (2018) that used the same sample of participants to study own-body-transformation (OBT) abilities. The authors found that, in the front facing orientation when OBT involved a mental rotation, OBT was very difficult for participants under 12 and the rates of OBT were similar among the three groups (ASD, DCD, and TD). Consequently, we explored imitation abilities regarding the three following aspects: interpersonal synchronization, participant's motor coordination, and control.

**Figure 3:**
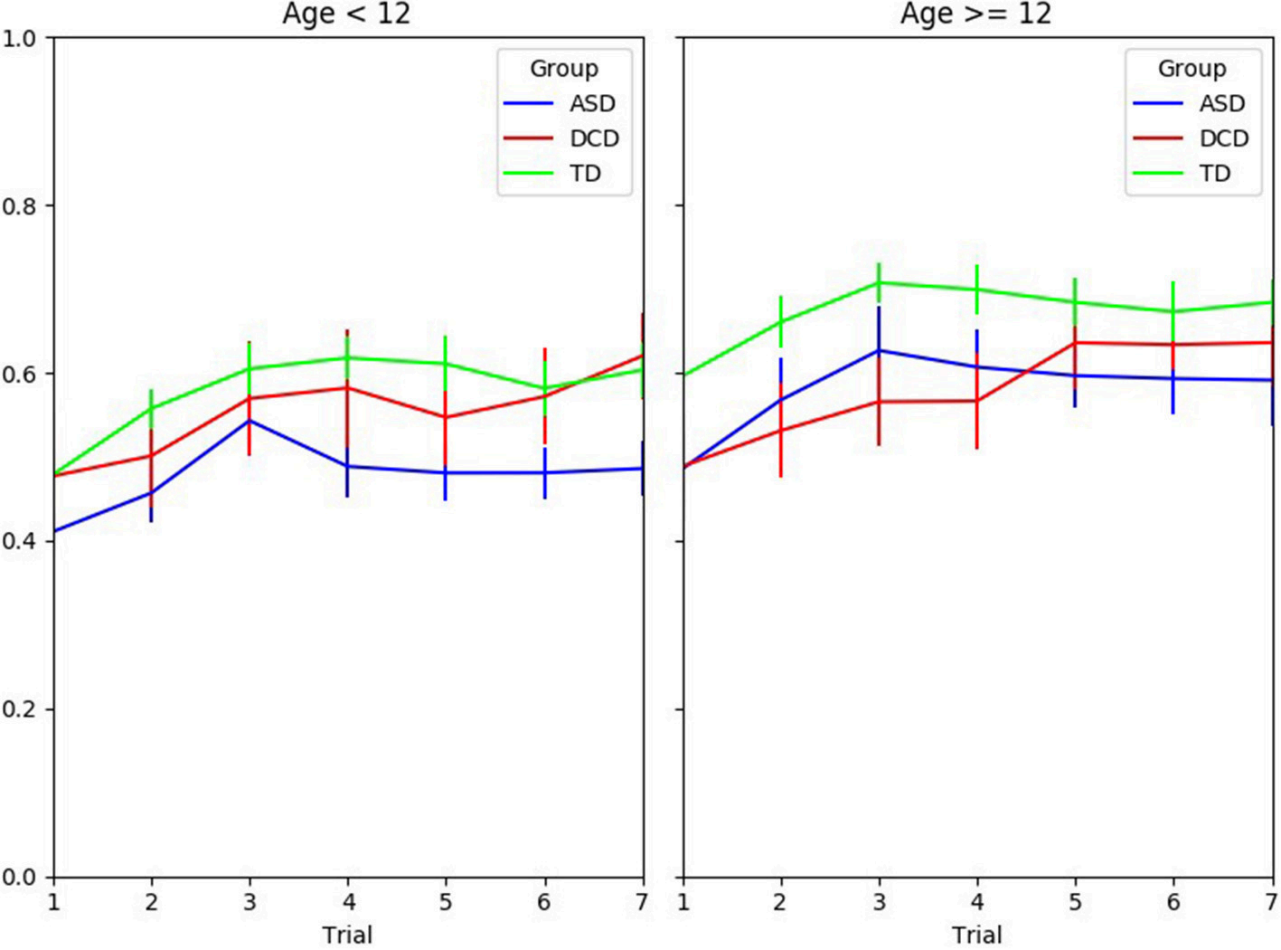
Correlation coefficient between the participant's bar and the tightrope walker's bar during the experiment according to groups and age.

Figure 3 shows the interpersonal synchronization of the interactive partners as measured by the correlation coefficients between the participant's bar and the tightrope walker's bar angles during the experiment according to groups and age. The GLMM model found several significant effects: correlation increased with age (β = 0.017, *p* < 0.001); correlation increased with the number of trials (β = 0.015, *p* < 0.001). There was also a significant effect according to groups: Correlation was smaller in the ASD group compared to both the TD group (β = −0.173, *p* < 0.001) and the DCD group (β = −0.124, *p* < 0.001).

**Figure 4:**
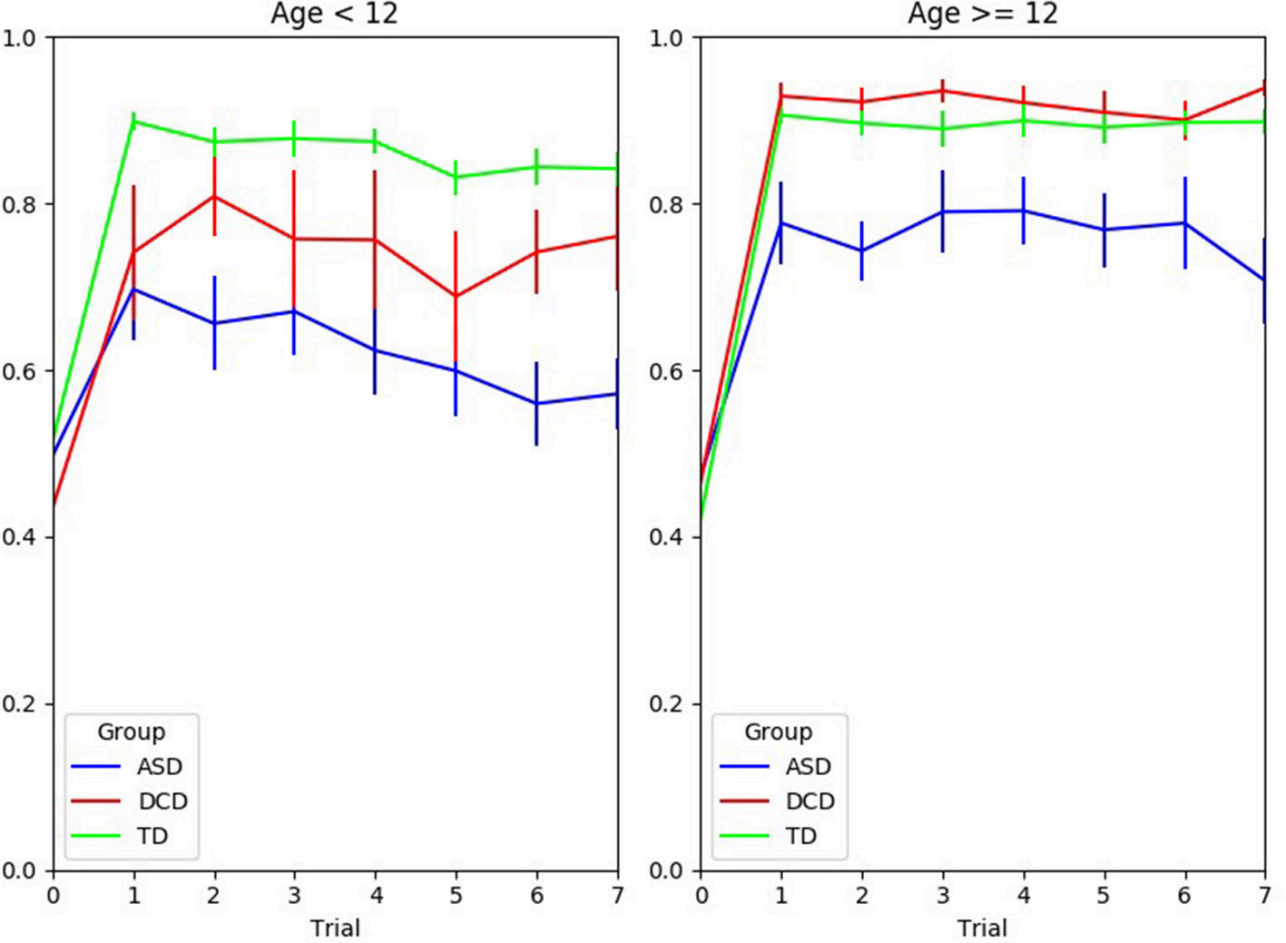
Correlation coefficient between the participant's bar and its head axis during the experiment according to groups and age.

Figure 4 shows the participants' motor coordination as measured by the correlation coefficient between the participant's bar and its head axis angles during the experiment according to groups and age. The GLMM model found several significant differences: the correlation increased with age (β = 0.019, *p* < 0.001), and with the number of trials (β = 0.006, *p* < 0.034). There was also a significant effect according to groups. Correlation was smaller in ASD compared to both the TD group (β = −0.27, *p* < 0.001) and the DCD group (β = −0.20, *p* < 0.001).

**Figure 5:**
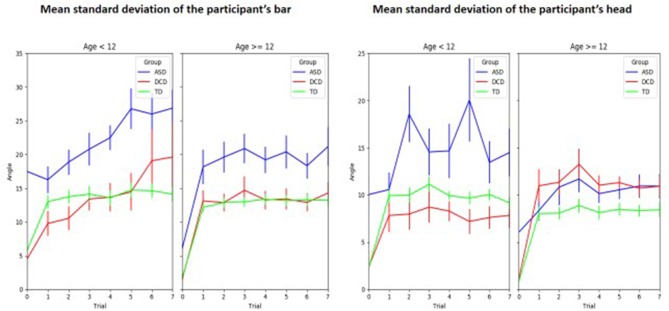
Mean standard deviations of the participant's bar and head movements during the experiment according to groups and age.

In terms of the participant's motor control, Figure 5 shows the standard deviations of the participant's bar and head angles during the experiment according to groups and age. For the participant's bar angle SD (Figure 4, left panel), the GLMM model found several significant effects. Mean SD decreased with age (β = −0.35, *p* = 0.029), increased with the number of trials (β = 1.2, *p* < 0.001). There was also a significant effect according to groups. Mean SD increased in ASD compared to TD (β = 7.14, *p* < 0.001) or compared to DCD (β = 7.15, *p* < 0.001). Results were very similar for the standard deviation of the participant's head angle (Figure 4, right panel). The GLMM model found several significant effects. Mean SD decreased with age (β = −0.26, *p* = 0.028), increased with the number of trials (β = 0.59, *p* < 0.001). There was also a significant effect according to groups. Mean SD increased in ASD compared to TD (β = 3.97, *p* < 0.001) or compared to DCD (β = 3.37, *p* < 0.001).

## Discussion

In this study, we explored the development of behavioral imitation abilities, in terms of interpersonal synchronization, motor coordination and control, and their respective potential impairments in ASD and DCD. To elucidate the contribution of visual spatial impairments and motor coordination impairments in ASD, we compared three groups: a group of children with ASD, a group of children with DCD and a group of TD children. We used a quantification of movements to analyze how motor performances evolved during the experimental task. To the best of our knowledge, it is the first clinical study on children assessing quality of interpersonal synchrony and coordination from a semi-ecological point of view through a spontaneous motor imitation task.

In TD children, we found a strong developmental change on behavioral imitation abilities as evidenced by the improvement of interpersonal synchronization (between the participant's bar and the tightrope walker's bar angles), motor coordination (between the participant's bar and its head axis angles), and control (standard deviation of the participant's bar movements, standard deviation of the participant's head), with age. This developmental effect is in line with current knowledge on imitation abilities that increase during child development (Rogers and Williams, 2006; Xavier et al., 2017). We also found a significant increase of interpersonal synchronization, motor coordination and control with the number of trials. This means that children improved during the task which is a common result in semi-ecological continuous task when the duration is short enough to keep child's motivation and avoid fatigability (Chambaron et al., 2006).

In patients with neuro-developmental disorders, the results were quite different. Although all patients were able to perform the task, the ASD group showed significantly lower behavioral imitation abilities compared to the TD group. These results are in line with several studies in literature (Sevlever and Gillis, 2010; Nadel, 2014; Vivanti and Hamilton, 2014). In the current study involving a continuous and meaningless task, our results are concordant with those of several authors arguing for lower performances in comparison to TD (specifically in goalless actions) (Edwards, 2014; Williams et al., 2014) contrary to preserved performances found when imitated actions have a visual goal (Gowen, 2012). Contrary to Young et al. (2011) who argues that imitation abilities are delayed and then could improve with age, our results before and after 12 years old, revealed that imitation in ASD is following a deviant development. Furthermore, the results confirmed those obtained by Gauthier et al. (2018) using the same sample which aimed to explore own-body transformation in ASD. On the basis of a manual annotation of children's movement trajectories, the authors found that the ASD group had a number of imitation responses to the TW movements significantly lower compared to the TD and DCD groups.

A specific input of the current study is the continuous automatic measures of participants and TW avatars that our computational setting allowed. We found lower performances in interpersonal synchrony in the ASD group in comparison with both TD and DCD groups which is concordant with impairments in interpersonal synchrony (Marsh et al., 2013; Fitzpatrick et al., 2016) and kinematic aspects of imitation (Vivanti and Hamilton, 2014) described in this population. In line with several authors (Mari et al., 2003; Schmitz et al., 2003; Rinehart et al., 2006; Fournier et al., 2010; Dowd et al., 2012; MacNeil and Mostofsky, 2012; Wild et al., 2012; Gowen and Hamilton, 2013), we found that the ASD individuals had lower performances in motor control, as shown by the large standard deviation of bar and head angle during the experiment (Figure 4) that significantly differed from that of both TD and DCD groups. Furthermore, motor control and interpersonal synchronization were higher in DCD patients compared to ASD patients independent of the other characteristics entered in the multivariate models.

Based on these results, we can argue that in ASD motor imitation impairments could not entirely be explained by visual spatial impairments and DCD comorbidity that are often described in ASD (Ghaziuddin and Butler, 1998; Pan et al., 2009; Fournier et al., 2010; MacNeil and Mostofsky, 2012). It is likely that the low performances found in ASD are related to impairments concerning the regulation of movement. In an ecological context close to ours, Fitzpatrick et al. (2013) investigated the dynamics of interactional synchronization in children and adolescents with ASD. Using a battery of imitation tasks (action sequences movements), they found that school-age children with ASD had lower social motor synchronization abilities than TD controls. In the same way, using a pendulum coordination paradigm, Fitzpatrick et al. (2016) found that adolescents with ASD performed worst on social motor synchronization tasks than TD controls. The authors argue that synchronization difficulties included in social motor coordination in ASD may be related to motor control impairments which manifested by more jerky and less accurate movements (Cook et al., 2013; Vivanti and Hamilton, 2014).

In our study, impairments in motor control found in ASD group are in accordance with those of several studies exploring motor interaction using robotic platforms. First, in a child/robot interaction built to induce joint attention, Anzalone et al. (2014) found a motor variability in the movements of both head and trunk in children with ASD. Boucenna et al. (2014, 2016) used a human-robot learning paradigm based on imitation of postures. They studied the impact of a human partner on the learning abilities of postures by a humanoid robot (Nao). They found that when Nao was interacting with participants, it was able to learn a social signature at the level of the group (meaning children with ASD as opposed to TD children) (Boucenna et al., 2014) but also at the level of individual recognition (Boucenna et al., 2016). In a seminal study, Guedjou et al. (2017) further showed that when Nao was interacting with children with ASD, the posture recognition was lower than that found after interacting with TD children. Altogether, the authors interpreted the specificity of Nao learning during motor imitation with children with ASD to a variability of the movement trajectories shown by these children.

Development of motor control requires forming an internal model of action relying on the coupling between action (motor commands) and perception (sensory feedback). Critical to the development of social, communicative, and motor coordination behaviors, internal model of action accurately predicts the sensory consequences of motor commands (Krakauer and Shadmehr, 2007). Thereupon, considering human brain organized by principles of Bayesian inferences and predictive coding, several authors recently proposed that autism may be a disorder of prediction (Van Boxtel and Lu, 2013; Gonzalez-Gadea et al., 2015; Bolis and Schilbach, 2017). This hypothesis of impaired prediction could potentially account for several significant correlates of autism as a reduced motor anticipation (see Sinha et al., 2014). In addition, interpersonal predictive coding incorporates time aspects (Manera et al., 2013) specifically highlighted in our setting using an ecologically valid real-time interaction. In that respect and according to Von der Lühe et al. (2016), impairments in imitation abilities with difficulties in interpersonal synchrony found in the ASD group are consistent with this hypothesis of predictive coding impairment in ASD.

Our results should be interpreted taking into consideration some limitations. First, the number of participants in the three groups was somewhat restricted, in particular in the DCD group compared to the two others. Second, visual attention abilities of the participants were not assessed despite their importance in performing the task. Finally, further studies could assess motor skills of ASD participants in accordance with DCD participants which would add consistency to the study confirming that, at the same motor abilities level, ASD group had more behavioral imitation impairments. However, it should be noted that, in the current study, motor control and motor coordination abilities involved in the imitation task correspond to milestones in very early child development, i.e., postural development in TD infants (André-Thomas and Ajuriaguerra, 1948; Lindsay et al., 2013).

## Conclusion

Behavioral imitation abilities during an ecological interaction showed subtle impairment in children with ASD as compared to children with TD or DCD, both in terms of interpersonal synchrony and motor coordination. These results questioned how motricity matures in terms of motor control and proprioception in children with ASD. Exploring motor control from a developmental point of view through a dynamic process like imitation poses significant pragmatic challenges for researchers and clinicians alike. In this regard, computational modeling involving human-machine interaction may be promising.

## Author contributions

All co-authors have participated in writing this manuscript. JX, DC, FV, MC, AB, and SA were involved in the introduction, discussion, and results parts of the manuscript. SG, SA, and MZ carried out the experimental part of our work (testing procedure and material). They participated in the writing of the description of the methodology of our work.

### Conflict of interest statement

The authors declare that the research was conducted in the absence of any commercial or financial relationships that could be construed as a potential conflict of interest.
